# A rare case of granuloma faciale presenting with ulceration: A case report

**DOI:** 10.1177/2050313X221093150

**Published:** 2022-04-24

**Authors:** Farhan Mahmood, Iris YH Teo, Steven J Glassman

**Affiliations:** 1 Faculty of Medicine, University of Ottawa, Ottawa, ON, Canada; 2 Department of Pathology and Laboratory Medicine, University of Ottawa, Ottawa, ON, Canada; 3 Division of Dermatology, Department of Medicine, University of Ottawa, Ottawa, ON, Canada

**Keywords:** Granuloma faciale, ulcer, monoclonal gammopathy, monoclonal gammopathy of undetermined significance

## Abstract

Granuloma faciale is an uncommon inflammatory dermatosis characterized by persistent dermal plaques, typically on the face, that mimic granulomatous disorders like sarcoidosis. Ulceration of granuloma faciale has very rarely been reported, and the plaques are usually asymptomatic and of cosmetic impact. We present a case of an 83-year-old male with recurrent granuloma faciale with spontaneous ulceration and monoclonal gammopathy of undetermined significance. Intralesional triamcinolone, 10 mg/mL monthly for 5 months, with pimecrolimus cream twice daily resolved the ulceration and the lesion continues to flatten and lighten. Ulceration is rare and atypical in granuloma faciale lesions which can be treated.

## Introduction

Granuloma faciale (GF) is an uncommon inflammatory dermatosis characterized by persistent dermal plaques, typically on the face, that mimic granulomatous disorders like sarcoidosis. Plaques are typically solitary reddish-brown to violaceous in color with follicular accentuation and telangiectases, ranging in size from 0.5 to 8 cm.^1,2^ Ulceration has been very rarely reported, and the plaques are usually asymptomatic and of cosmetic impact. Associations are uncommon and include IgG4-related diseases and monoclonal gammopathy. Lesions are often recalcitrant despite several modalities of treatment, with a high tendency to recurrence.^1–3^ The etiology of GF is unclear; it has been considered a form of chronic leukocytoclastic vasculitis. Actinic damage, allergy, trauma, and radiation therapy have also been implicated in the pathogenesis of GF.^1,2,4^ A case of recurrent GF with spontaneous ulceration is presented in an 83-year-old man with monoclonal gammopathy.

## Case report

An 83-year-old man describes a lesion on the central forehead for 5 years. There was no preceding trauma, and the lesion was mostly asymptomatic but of aesthetic concern. Following two excisions by plastic surgery, there was a recurrence and the development of a spontaneous ulcer within, which was very tender. He was otherwise well and on no medication.

**Figure 1.**
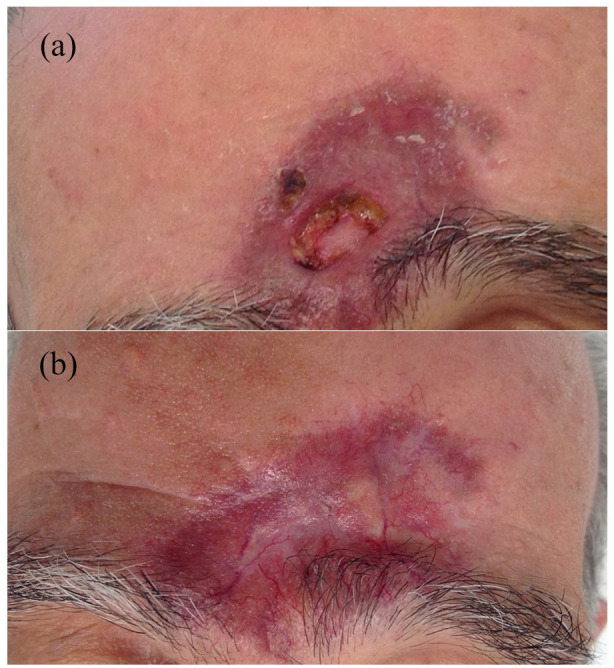
(a) Solitary lesion of GF with ulcer on the central forehead. (b) Lesion of GF after successful treatment of the ulcer with a combination of intralesional triamcinolone and pimecrolimus cream.

The patient was noted to be a well-looking man with Fitzpatrick type III skin. In the glabellar region, there was a solitary 7 cm×5 cm brown-purple, shiny, bossellated, annular, firm plaque with large overlying telangiectasias (Figure 1(a)). There was a 1-cm deep ulcer centrally but no cervical lymphadenopathy.

**Figure 2.**
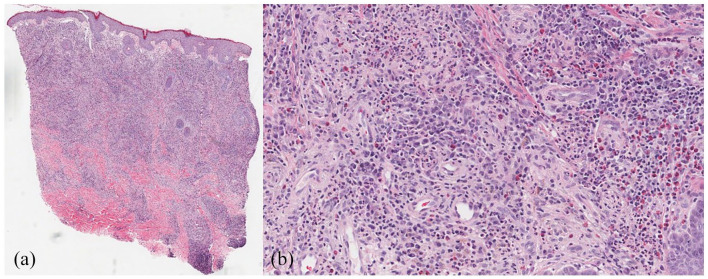
(a) A diffuse dermal infiltrate extends into the deep dermis, occasionally accompanying adnexal structures. A grenz zone is present. Note the superficial dilated lymphatics (10× magnification, H&E stain). (b) Higher magnification reveals a mixed population of plasma cells, neutrophils, eosinophils, lymphocytes, and histiocytes. Most vessels are dilated and are lined by plump endothelial cells (200× magnification, H&E stain).

Skin biopsy showed inflammation involving the entire dermis composed of lymphocytes, plasma cells, histiocytes, neutrophils, and eosinophils (Figure 2). There was a grenz zone, and the epidermis showed mild spongiosis. The vessels showed marked swelling of endothelial cells. Tissue culture for bacterial, fungal, and mycobacterial infection was negative. The clinical and pathologic features were typical of GF. The complete blood count and differential was normal. Serum protein electrophoresis showed a monoclonal gammopathy IgG Kappa, which was diagnosed as monoclonal gammopathy of undetermined significance (MGUS) by a hematologist. IgG4 level was normal. Creatinine and alanine transaminase were normal.

The patient was treated with intralesional triamcinolone, 10 mg/mL monthly for 5 months, together with pimecrolimus cream twice daily. The ulcer healed and the lesion continues to flatten and lighten gradually but is still present (Figure 1(b)). Consent was provided by the patient to publish this case.

## Discussion

To our knowledge, there are only two cases previously reported of ulceration in a GF lesion. A retrospective analysis of 66 patients diagnosed with GF (mean age of 53 years and 62% males) previously found one case of GF with ulceration and two cases presenting with annular lesions; the majority of GF lesions were plaques or nodules.^3^ In addition to ulcers, other atypical presentations included yellowish lesions, Darier’s sign and possibly Koebner’s phenomenon.^3^ Deen et al.^5^ described a well-demarcated violaceous plaque in the right preauricular region with central ulceration in a 63-year-old white male. The lesion was successfully treated with intralesional corticosteroids. The patient had a past medical history of indolent lymphoma with monoclonal gammopathy.

Although the pathogenesis of GF is unclear, a form of chronic vasculitis mediated by a localized Arthus-like reaction has been postulated. GF has been shown to be associated with clonally expanded populations of CD4+ lymphocytes, IL-5 production, infiltrates of IgG4+ plasma cells, and vascular inflammation.^6,7^ GF lesions have also recently been proposed to share clinical features with localized forms of IgG4-related sclerosing diseases, with abnormal levels of IgG4+ plasma cells.^7^ According to the consensus statement on the pathology of IgG4-related diseases, major histopathological features associated with IgG4-related diseases include a dense lymphoplasmacytic infiltrate, fibrosis arranged in a storiform pattern, and obliterative phlebitis,^8^ all of which may be observed in GF. However, Kavand et al.^9^ demonstrate that there is a lack of evidence to definitively associate GF with IgG4-related diseases. This study also highlights an association between monoclonal gammopathy (which was diagnosed as MGUS in the present case) and erythema elevatum diutinum (EED), a rare dermatosis postulated to have a similar underlying pathogenesis to GF. In addition to EED, other clinical differential diagnoses for GF include sarcoidosis, discoid lupus erythematosus, granulomatous rosacea, mycobacterial infections, deep fungal infections, cutaneous lymphoma, and basal cell carcinoma.^1^

Treatment of GF can be very challenging. Surgical excision, cryosurgery, or laser therapy have shown variable success.^4^ A case of GF on the transconjunctival surface of the eyelid was fully excised without any recurrence.^10^ Other treatment modalities include topical and intralesional corticosteroids, topical calcineurin inhibitors, topical and systemic dapsone, clofazimine, and tumor necrosis factor-α inhibitors.^1,3^
